# Simultaneously improving xylose fermentation and tolerance to lignocellulosic inhibitors through evolutionary engineering of recombinant *Saccharomyces cerevisiae* harbouring xylose isomerase

**DOI:** 10.1186/1472-6750-14-41

**Published:** 2014-05-15

**Authors:** Justin Smith, Eugéne van Rensburg, Johann F Görgens

**Affiliations:** 1 Department of Process Engineering, Stellenbosch University, Private Bag X1, Matieland 7602, South Africa

**Keywords:** *Saccharomyces cerevisiae*, Yeast hardening, Evolutionary engineering, Random mutagenesis, Triticale hydrolysate, EMS, Lignocellulose

## Abstract

**Background:**

Yeasts tolerant to toxic inhibitors from steam-pretreated lignocellulose with xylose co-fermentation capability represent an appealing approach for 2^nd^ generation ethanol production. Whereas rational engineering, mutagenesis and evolutionary engineering are established techniques for either improved xylose utilisation or enhancing yeast tolerance, this report focuses on the simultaneous enhancement of these attributes through mutagenesis and evolutionary engineering of *Saccharomyces cerevisiae* harbouring xylose isomerase in anoxic chemostat culture using non-detoxified pretreatment liquor from triticale straw.

**Results:**

Following ethyl methanesulfonate (EMS) mutagenesis, *Saccharomyces cerevisiae* strain D5A^+^ (ATCC 200062 strain platform), harbouring the xylose isomerase (XI) gene for pentose co-fermentation was grown in anoxic chemostat culture for 100 generations at a dilution rate of 0.10 h^-1^ in a medium consisting of 60% (v/v) non-detoxified hydrolysate liquor from steam-pretreated triticale straw, supplemented with 20 g/L xylose as carbon source. In semi-aerobic batch cultures in the same medium, the isolated strain D5A^+H^ exhibited a slightly lower maximum specific growth rate (μ_max_ = 0.12 ± 0.01 h^-1^) than strain TMB3400, with no ethanol production observed by the latter strain. Strain D5A^+H^ also exhibited a shorter lag phase (4 h vs. 30 h) and complete removal of HMF, furfural and acetic acid from the fermentation broth within 24 h, reaching an ethanol concentration of 1.54 g/L at a yield (Y_p/s_) of 0.06 g/g xylose and a specific productivity of 2.08 g/gh. Evolutionary engineering profoundly affected the yeast metabolism, given that parental strain D5A^+^ exhibited an oxidative metabolism on xylose prior to strain development.

**Conclusions:**

Physiological adaptations confirm improvements in the resistance to and conversion of inhibitors from pretreatment liquor with simultaneous enhancement of xylose to ethanol fermentation. These data support the sequential application of random mutagenesis followed by continuous culture under simultaneous selective pressure from inhibitors and xylose as primary carbon source.

## Background

*Saccharomyces cerevisiae* remains the preferred microbe for producing ethanol from pretreated lignocellulose, given its general robustness, high ethanol production rates and ethanol tolerance [1-3]. Critical interventions required to enhance the efficiency of this yeast for commercial 2^nd^ generation ethanol production include (i) introducing capability to ferment xylose [1,4,5] and (ii) enhancing tolerance to toxic by-products from steam pretreatment [1,6-9].

Two approaches can be followed for introducing xylose utilising capability in *S. cerevisiae*, namely the cloning of xylose reductase (XR) and xylitol dehydrogenase (XDH) or cloning xylose isomerase (XI), usually in conjunction with xylulokinase (XK) to convert xylulose to xylulose-5-phosphate. These rational metabolic engineering approaches were exemplified in the respective benchmark studies of Wahlbom *et al*. [10] and Kuyper *et al*. [11]. Other examples where rational approaches were followed for improved xylose metabolism include studies by Gorsich *et al*. [12] and Toivari *et al*. [13]. Enhancing yeast tolerance, or “hardening” requires a more comprehensive intervention employing rational approaches to genome modification, random mutagenesis and directed evolutionary engineering under selective pressure (see excellent reviews by Sauer [14] and Nevoigt [15]). It should be noted, however, that these techniques can also be employed to enhance xylose utilisation in the absence of hydrolysate inhibitors (see below), which suggests technique overlap in achieving both improved xylose utilisation and inhibitor tolerance. Furthermore, rational approaches are often challenging due to the complexity of the yeast genome, requiring an in-depth understanding of the metabolome and its response to dynamic inputs, making the investigator reliant on a variety of “–omics” technologies [15]. Rational approaches were also occasionally found insufficient to instil required capabilities in the yeast phenotype [16]. As such, random mutagenesis and selection followed by evolutionary engineering is often the methodological sequence of choice, where the former could lead to phenotypes with enhanced capabilities for either xylose utilisation or inhibitor tolerance but without prior knowledge of specific metabolic pathways. The latter allows for selection under process-relevant conditions [14], especially where inhibitors from lignocellulosic pretreatment are present.

In the literature, strain development predominantly focused on the XR/XDH/XK system where strains with the TMB prefix, produced by Swedish researchers, featured quite prominently. *S. cerevisiae* TMB3400 produced by Wahlbom *et al*. [10] was later subjected to evolutionary engineering using furfural in sequential batch culture [17], and in another study to sequential batch and continuous culture at a dilution rate (D) of 0.05 h^-1^ using spruce hydrolysate, subsequent to UV mutagenesis [18]. Similarly, *S. cerevisiae* strain TMB3001 [19] also harbouring XR/XDH/XK was subjected to evolutionary engineering in continuous culture (D = 0.05 h^-1^) in the absence of inhibitors to enhance xylose fermentation [20], as well as in continuous culture (D = 0.1 h^-1^) in the presence of hydrolysate [21,22]. Other strains of *S. cerevisiae* were also subjected to either mutagenesis [23] or evolutionary engineering [24,25] or both [26] (all in the absence of inhibitors), with a few instances where other yeast species were also investigated for enhanced xylose utilisation, including *Scheffersomyces* (*Pichia*) *stipitis*[27] and *Pachysolen tannophilus*[28]. Amartey and Jeffries [29] described enhanced tolerance by a *Pichia* strain after sequential subculture in different concentrations of corn cob hydrolysate, after overliming with Ca(OH)_2_ and in an innovative approach, Almario *et al*. [30] used visualising evolution in real-time (VERT) to assess molecular mechanisms associated with short term adaptation [31] in batch culture.

Noteworthy studies pertaining to strain development harbouring the xylose isomerase (XI) only appeared after the successful expression of XI from *Piromyces* in *S. cerevisiae*[11] followed by evolutionary engineering in oxygen-limited chemostat culture at D = 0.06 h^-1^ for 2 000 hours in the absence of hydrolysate. Similar work was published by Wisselink *et al*. [32] and Zhou *et al*. [33] using sequential batch and continuous culture. In these studies, cassettes containing several enzymes from the pentose phosphate pathway and for arabinose utilisation were transformed into *S. cerevisiae*. However, in none of these studies were hydrolysates used as additional selective pressure criterion for evolutionary engineering of the xylose fermenting yeast in continuous culture. In fact, to the knowledge of the authors, there appeared only one study where close to the full range of strain development techniques was applied to an XI-containing strain [16]. Using *S. cerevisiae* Ethanol Red as platform, the by now classical approach commenced with rational metabolic engineering through transformation with a cassette containing the XI gene from *Clostridium phytofermentans*, followed by random mutagenesis using EMS with hydrolysate and xylose as selective criteria. Gene shuffling preceded evolutionary engineering carried out in sequential batch culture without hydrolysate but at very high concentrations of xylose (40 g/L). Using a mating approach in a follow-up study [34], this strain designated GS1.11-26 was mated with another tolerant yeast strain after screening 580 yeast strains using dilute acid-pretreated spruce hydrolysate. Three resulting strains, designated as GSF335, GSF767 and GSE16 revealed a marked improved phenotype compared to Ethanol Red, producing up to 23% (v/v) more ethanol.

The purpose of the present study was to improve the inhibitor tolerance and simultaneously enhance xylose fermentation to ethanol of a recombinant xylose-utilising *S. cerevisiae* strain D5A^+^ harbouring the *xylA* gene from *Bacteriodes thetaiotaomicron* producing XI. “Hardening” was accomplished through a combination of random mutagenesis with EMS and long-term evolutionary engineering in chemostat culture using xylose as carbon source and liquor from steam-pretreated triticale straw as selective criteria at both steps (mutagenesis and chemostat culture). The degree of hardening achieved was evaluated through comparison of the fermentative performance of the hardened yeast to the original parental strain, during exposure to pretreatment liquor supplemented with either glucose or xylose in batch culture. Two additional non-recombinant *S. cerevisiae* strains MEL2 and MH1000, as well as *S. cerevisiae* strain TMB3400, were included for comparison. Finally, the fermentative performance of the hardened yeast under SSF conditions was assessed using pressed steam-pretreated sweet sorghum bagasse as substrate.

## Results

### Chemical characterisation of steam pretreated triticale straw and sweet sorghum bagasse

The chemical composition of the liquor from steam-pretreated triticale straw (used in continuous culture) and sweet sorghum bagasse (used in SSF experiments) are shown in Table 1. The transition between the two feedstocks was required due to limited material availability. Xylose was the most abundant sugar in both the triticale and sorghum pretreatment liquor fractions. This result supported the general observation that predominantly hemicellulose is solubilised during steam pretreatment [35,36] although it should be noted that no acidic catalyst was used during pretreatment in the present study. After steam pretreatment the WIS fractions were first subjected to complete acid hydrolysis before chemical analysis. Glucose was the predominant sugar in the hydrolysed sweet sorghum WIS fraction, which was expected since the WIS fraction of the pretreated material was enriched for both lignin and cellulose, as previously documented [37,38]. Acetic acid was the most abundant by-product from steam-pretreatment, followed by furfural and HMF, in both the triticale and sweet sorghum pretreatment liquor fractions. Although the acetic acid concentration was significantly greater (17%) in the sorghum pretreatment liquor, the sugar degradation products furfural and HMF were 1.5 and 1.8 times more concentrated in the triticale pretreatment liquor. Phenolic compounds and their respective degradation products were not quantified in this study. Given that HMF, furfural and acetic acid are the main by-products responsible for inhibition of microbial metabolism [39], specific emphasis was placed on the concentration of these by-products as an indicator of pretreatment liquor toxicity.

**Table 1:** Chemical composition of steam-pretreated triticale straw and sweet sorghum bagasse

	**Triticale pretreatment liquor (g/L)**	**Sweet sorghum pretreatment liquor (g/L)**	**Sweet sorghum water-insoluble solids (g/100 g)**
**By-products**			
Acetic acid	3.34 ± 0.10	4.01 ± 0.30	-
Formic acid	0.94 ± 0.10	0.72 ± 0.20	-
HMF	0.44 ± 0.01	0.23 ± 0.02	-
Furfural	1.63 ± 0.05	1.08 ± 0.05	-
**Sugars**			
Glucose	0.71 ± 0.05	0.27 ± 0.03	51.48 ± 0.3
Xylose	3.24 ± 0.50	2.70 ± 0.06	12.29 ± 0.23
Cellobiose	0.16 ± 0.01	0.59 ± 0.01	10.49 ± 1.22
Arabinose	0.24 ± 0.02	0.67 ± 0.09	0.46 ± 0.10

Conditions of steam pretreatment for triticale straw and sweet sorghum bagasse were 200°C for 20 min and 180°C for 5 min, respectively. The chemical composition of the water insoluble solid fraction was determined after washing and subsequent to acid hydrolysis. The composition of pretreated triticale straw WIS was excluded from the table since this fraction was not utilised in this study. Standard deviations from duplicate analyses are also shown.

### Random mutagenesis

**Figure 1:**
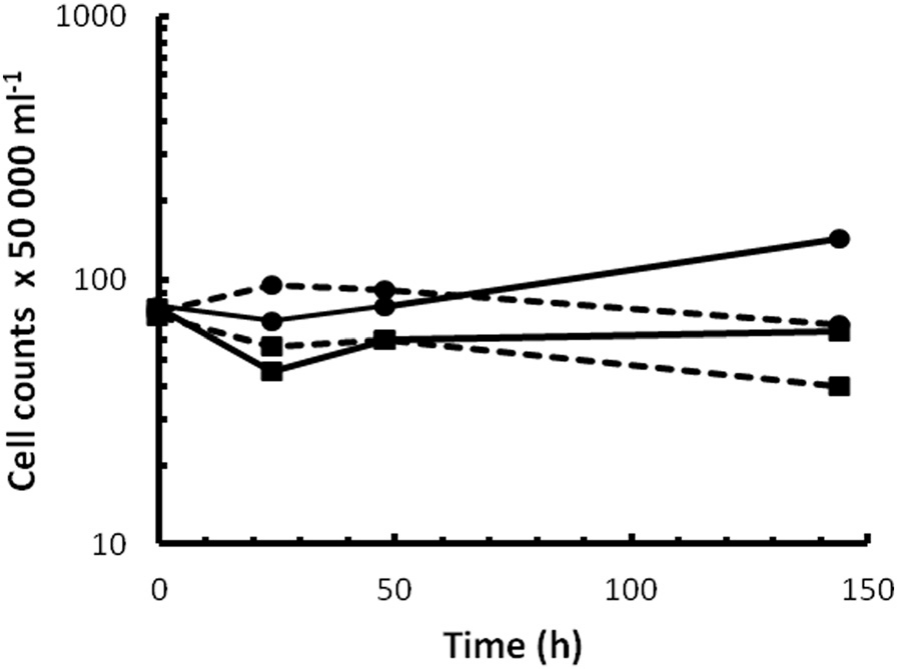
**Comparison of parental and mutated strain in batch culture.** Batch growth profiles for the *S. cerevisiae* parental strain D5A^+^ (squares) and mutant strain D5A^+^ (circles) in water-diluted (67% v/v, solid line) and undiluted (broken line) liquor from steam-pretreated triticale supplemented with 20 g xylose/L in shake flask cultures. Culture growth was quantified as total cell counts using a counting chamber. Data represents the average values from duplicate counts.

Yeast cells that survived chemical mutagenesis after exposure to 2 and 3 μl EMS/mL for 1 h were pooled and the growth compared to that of the parental D5A^+^ strain in a chemically-defined medium supplemented with diluted (67% v/v) and undiluted triticale pretreatment liquor with 20 g/L xylose as carbon source (Figure 1). Xylose in combination with the pretreatment liquor was essential for selecting mutants displaying functional xylose utilisation together with increased inhibitor tolerance. As evident from Figure 1, mutants of strain D5A^+^ reached substantially greater total cell counts (±2-fold greater) than the parental D5A^+^ strain in the diluted liquor, whereas the undiluted liquor resulted in decreased cell counts, possibly resulting from cell lysis due to the harshness of the medium. The dark medium colour precluded the use of turbidity to quantify culture growth, necessitating the use of serial dilutions and plate counts. Based on this data, a triticale hydrolysate concentration of 67% (v/v) was regarded as the maximum sub-lethal concentration to which the yeast could be exposed.

### Evolutionary engineering using continuous culture

Mutated cells were subjected to evolutionary engineering using continuous culture at an initial dilution rate of 0.05 h^-1^ for one week (Table 2). This low initial dilution rate would allow adaptation of cells to a relatively high pretreatment liquor concentration of 50% (v/v) while maintaining selective pressure, albeit at pretreatment liquor levels lower than the maximum sub-lethal concentration. Plate counts were used to confirm that the culture did not wash out of the reactor, upon which the dilution rate was incrementally adjusted over a further one week period to a dilution rate of 0.1 h^-1^. This dilution rate was maintained at a hydrolysate concentration of 50% (v/v) for 49 generations (two weeks), after which the hydrolysate concentration was increased to 60% (v/v) for a further 100 generations (four weeks). Substantial washout of the culture occurred at dilution rates greater than 0.125 h^-1^, which approximated the μ_max_ value of 0.12 h^-1^, recorded in batch culture (see below). Although attempts were made to attain steady state at a dilution rate close to 0.125 h^-1^, the proximity of this dilution rate to the critical dilution rate resulted in large variation in cell counts over a two week period at a hydrolysate concentration of 60% (v/v, Table 2). The absence of total wash-out could possibly be attributed to substantial wall growth after continuous growth for several weeks. Due to constraints in pretreatment liquor availability, no further increases in the concentration above 60% (v/v) were attempted. The structure of the population was not characterised, where a single colony was isolated after two rounds of subculture on YPX agar (in the absence of hydrolysate inhibitors) and used for subsequent work. This strategy was based on the premise that after 220 generations (Table 2) under stringent selective conditions (high inhibitor concentration, xylose as primary carbon source), the culture would be predominantly comprised of strains with improved performance and hence, a greater probability of isolating a more resistant strain. As the results showed, this was indeed the case. Developing an appropriate screening procedure of isolates with beneficial phenotypes is an ongoing activity in our group.

**Table 2:** Cell counts at steady state

**Dilution rate (h**^**-1**^**)**	**CFU/mL**	**Pretreatment liquor (%, v/v)**	**Time (weeks)**	**Generations**
Inoculation	1.8 × 10^6^ ± 0.42 × 10^6^	50	0	-
0.05	3.5 × 10^7^ ± 7.8 × 10^6^	50	1	13
0.1	38 × 10^6^ ± 4.8 × 10^6^	50	2	49
0.1	36 × 10^6^ ± 6.4 × 10^6^	60	4	97
0.125	41 × 10^6^ ± 17 × 10^6^	60	2	61
Total generations				220

Steady state biomass concentrations, expressed as plate counts (CFUs/mL) for the mutated strain D5A^+^ at increasing dilution rates with the corresponding time at which the chemostat was operated at each dilution rate. The biomass concentration at the time of inoculation is shown for comparison. Standard deviations represent triplicate determinations corresponding to measurements at three consecutive residence times.

### Analysis of strain performance in semi-aerobic batch culture

**Figure 2:**
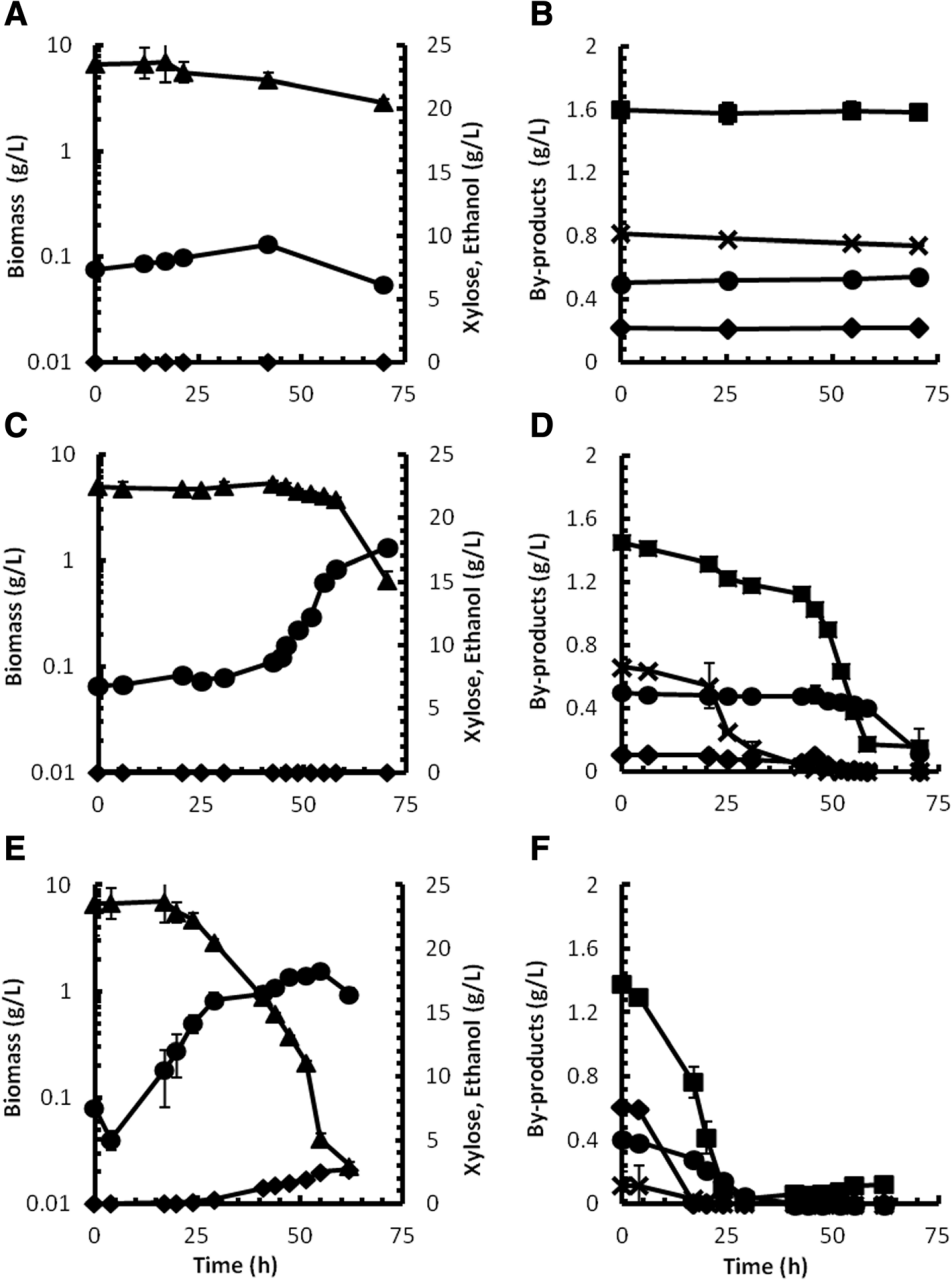
**Batch culture profiles after hardening with xylose as carbon source.** Batch culture profiles for the recombinant xylose-utilising *S. cerevisiae* strains D5A^+^ (parental strain, **A** and **B**), TMB3400 **(C and D)** and D5A^+H^ (hardened strain, **E** and **F**) in a growth medium supplemented with 50% (v/v) pretreatment liquor from steam-pretreated triticale and 20 g xylose/L in shake flask cultures. Figure symbols left-hand column: biomass (circles), ethanol (diamonds), xylose (triangles). Figure symbols right-hand column: acetic acid (squares), formic acid (circles), HMF (diamonds) and furfural (crosses). Error bars represent standard deviations from duplicate experiments.

**Figure 3:**
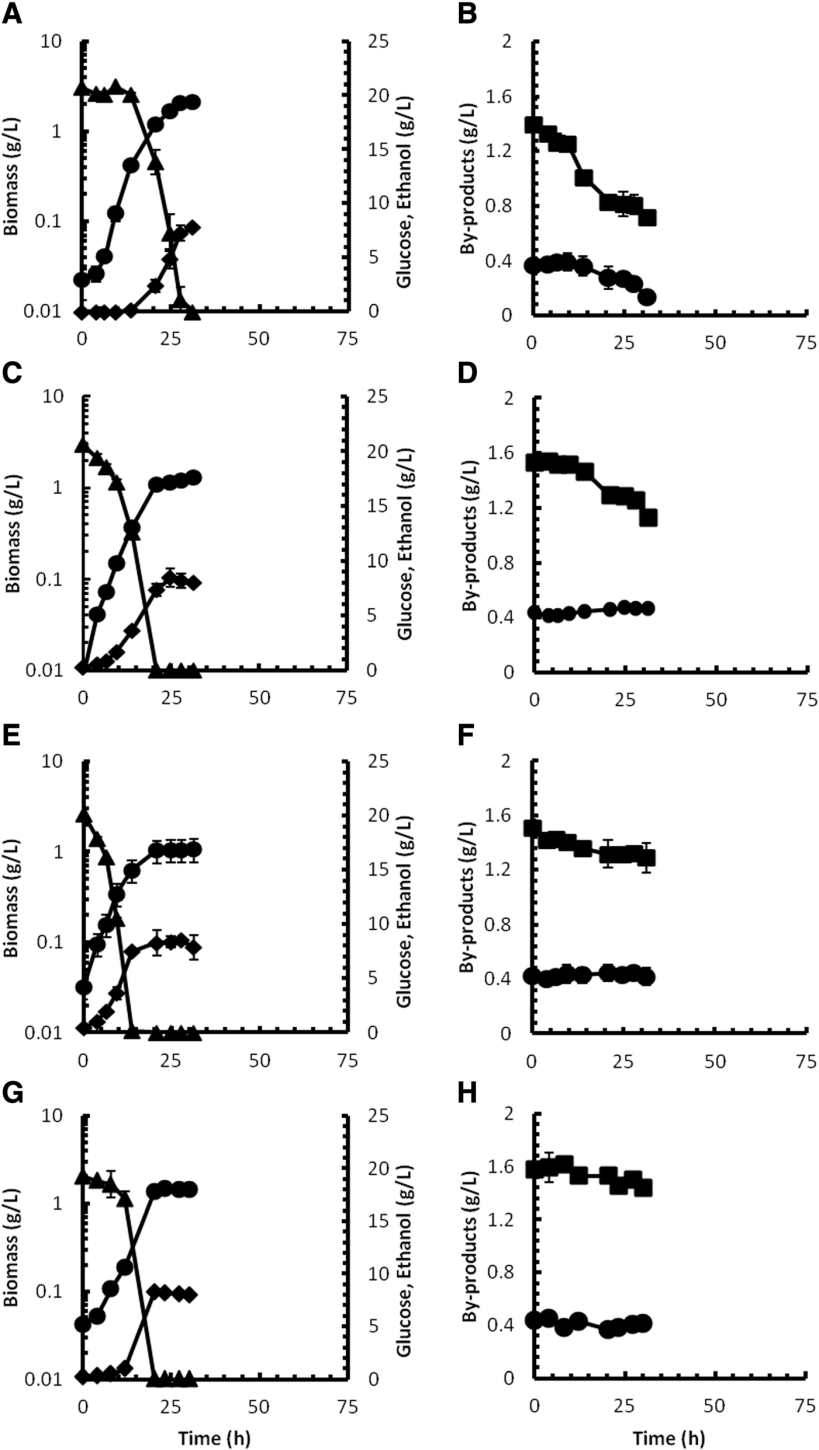
**Batch culture profiles after hardening with glucose as carbon source.** Batch cultivation profiles for *S. cerevisiae* strains D5A^+H^**(A and B)**, TMB3400 **(C and D)**, MH1000 **(E and F)** and MEL2 **(G and H)** in 50% (v/v) triticale pretreatment liquor supplemented with 20 g glucose/L in shake flask cultures. Figure symbols: biomass (circles), glucose (triangles), ethanol (diamonds), acetic acid (squares), formic acid (circles). Error bars represent standard deviations from duplicate experiments.

The strain isolated from continuous culture after 100 generations was designated as strain D5A^+H^ and was grown in batch culture on either xylose or glucose to assess its ability to utilise these carbon sources in the presence of pretreatment liquor. Culture growth was also compared to three other strains of *S. cerevisiae* namely strains MEL2 [40], MH1000 [41] and TMB3400 [10], also supplemented with liquor from steam-pretreated triticale. Strain D5A^+^ (parental recombinant strain not subjected to mutagenesis) was included as control on both carbon sources in the presence of liquor. As additional control, the parental D5A^+^ strain was also grown in the defined medium without hydrolysate, with either xylose or glucose as carbon source. In all other experiments the different strains were grown at a pretreatment liquor concentration of 50% (v/v). Fermentation profiles of the recombinant xylose-utilising yeast grown on xylose are shown in Figure 2, with the fermentation profiles on glucose shown in Figure 3. Tables 3 and 4 provide a summary of fermentation parameters calculated from duplicate batch experiments.

**Table 3:** Growth parameters with xylose as carbon source

**Parameter**	**No liquor**	**Supplemented with liquor**		
	**D5A**^**+**^	**D5A**^**+**^	**D5A**^**+H**^	**TMB3400**
μ_max_ (h^-1^)	0.37 ± 0.002	N/D	0.12 ± 0.01	0.14 ± 0.01
Biomass^†^ (g/L)	2.08 ± 0.01	N/D	0.82 ± 0.1	0.84 ± 0.01
Ethanol^†^ (g/L)	N/D	N/D	1.54 ± 0.14	N/D
Glycerol^†^ (g/L)	0.15 ± 0.01	N/D	2.7 ± 0.27	0.19 ± 0.05
Y_x/s_^‡^ (g/g)	0.55 ± 0.023	N/D	0.07 ± 0.001	0.51 ± 0.06
Y_p/s_^§^ (g/g)	N/D	N/D	0.06 ± 0.001	N/D
Y_p/x_^¶^ (g/g)	N/D	N/D	1.9 ± 0.03	N/D
q_s_^*^ (g/gh)	0.17 ± 0.003	N/D	2.08 ± 0.05	0.23 ± 0.02
q_p_^††^ (g/gh)	N/D	N/D	0.02 ± 0.003	N/D

Yield coefficients and specific rates of growth, substrate consumption and ethanol production for strains D5A^+^, D5A^+H^ and TMB3400 with 20 g xylose/L as primary carbon source in batch culture with and without with 50% (v/v) hydrolysate liquor from steam-pretreated triticale. Standard deviations from duplicate fermentations are shown.

N/D None detected.

^†^Biomass and product concentrations at the end of the exponential growth phase.

^‡^Y_x/s_, biomass yield on xylose, calculated from the slope of the biomass concentration plotted as a function of the residual xylose concentration.

^§^Y_p/s_, ethanol yield on xylose, calculated from the slope of the ethanol concentration plotted as a function of the residual xylose concentration.

^¶^Y_p/x_, ethanol yield on biomass, calculated from the slope of the ethanol concentration plotted as a function of the biomass concentration.

^*^q_s_, specific rate of substrate utilisation in g xylose/g biomass per hour during the mid-exponential phase.

^††^q_p_, specific rate of product formation in g ethanol/g biomass per hour during the mid-exponential phase.

**Table 4:** Growth parameters with glucose as carbon source

**Parameter**	**No liquor**	**Supplemented with liquor**				
	**D5A**^**+**^	**D5A**^**+**^	**D5A**^**+H**^	**TMB3400**	**MEL2**	**MH1000**
μ_max_ (h^-1^)	0.56 ± 0.01	0.03 ± 0.01	0.29 ± 0.02	0.22 ± 0.00	0.21 ± 0.01	0.23 ± 0.01
Biomass^†^ (g/L)	1.42 ± 0.06	1.18 ± 0.05	2.13 ± 0.04	1.32 ± 0.02	1.46 ± 0.02	1.07 ± 0.02
Ethanol^†^ (g/L)	7.77 ± 0.15	8.86 ± 0.30	7.76 ± 0.13	8.48 ± 0.14	8.28 ± 0.02	8.55 ± 0.50
Glycerol^†^ (g/L)	0.67 ± 0.01	0.44 ± 0.00	0.51 ± 0.01	0.46 ± 0.01	0.39 ± 0.07	0.43 ± 0.06
Y_x/s_^‡^ (g/g)	0.08 ± 0.00	0.06 ± 0.01	0.1 ± 0.00	0.06 ± 0.01	0.08 ± 0.00	0.05 ± 0.00
Y_p/s_^§^ (g/g)	0.42 ± 0.01	0.43 ± 0.03	0.37 ± 0.01	0.42 ± 0.01	0.43 ± 0.00	0.44 ± 0.02
Y_p/x_^¶^ (g/g)	5.18 ± 0.03	5.37 ± 0.2	3.58 ± 0.2	6.16 ± 0.7	6.08 ± 0.06	6.85 ± 0.3
q_s_^*^ (g/gh)	3.66 ± 0.07	1.6 ± 0.14	1.1 ± 0.1	3.03 ± 0.1	4.64 ± 0.38	6.4 ± 0.1
q_p_^††^ (g/gh)	2.25 ± 0.00	0.69 ± 0.00	0.41 ± 0.00	0.54 ± 0.03	0.85 ± 0.03	1.8 ± 0.02

Yield coefficients and specific rates of growth, substrate consumption and ethanol production for strains D5A^+^, D5A^+H^, TMB3400, MH1000 and MEL2 with 20 g glucose/L as primary carbon source in batch culture with and without with 50% (v/v) hydrolysate liquor from steam-pretreated triticale. Standard deviations from duplicate fermentations are shown.

^†^Biomass and product concentrations at the end of the exponential growth phase.

^‡^Y_x/s_, biomass yield on glucose, calculated from the slope of the biomass concentration plotted as a function of the residual glucose concentration.

^§^Y_p/s_, ethanol yield on glucose, calculated from the slope of the ethanol concentration plotted as a function of the residual glucose concentration.

^¶^Y_p/x_, ethanol yield on biomass, calculated from the slope of the ethanol concentration plotted as a function of the biomass concentration.

^*^q_s_, specific rate of substrate utilisation in g glucose/g biomass per hour during the mid-exponential phase.

^††^q_p_, specific rate of product formation in g ethanol/g biomass per hour during the mid-exponential phase.

In the presence of 50% pretreatment liquor with 20 g/L xylose as carbon source, strain D5A^+H^ displayed a marked improvement in terms of culture growth compared to the control strain as evident from a more than 10-fold increase in biomass concentration (Figure 2E). Strain D5A^+H^ also exhibited a significantly shorter lag phase of less than 4 h compared to that of strain ТMB3400 (Figure 2C), which suggested that strain D5A^+H^ exhibited a greater level of tolerance towards the inhibitors in the growth medium. However, there was no significant difference between the μ_max_ values of strains D5A^+H^ and ТMB3400 (Table 3). The performance of strain D5A^+H^ stood in stark contrast to the poor growth and xylose utilisation of strain D5A^+^ (parental strain, not subjected to hardening, Figure 2A) in the presence of inhibitors. Furthermore, in the absence of inhibitors, the parental D5A^+^ strain produced no ethanol, whereas an ethanol concentration of up to 1.54 g/L at an ethanol yield (Y_p/s_) of 0.06 g/g xylose was recorded for strain D5A^+H^ in the presence of inhibitors. Although low levels of glucose was present in the hydrolysate from pretreatment (0.71 g/L, Table 1), this low concentration was insufficient to support an ethanol titre of 1.54 g/L (Table 3), pointing to improved ethanol fermentation from xylose by this strain subsequent to mutagenesis and evolutionary engineering.

Xylose consumption by strain D5A^+H^ closely followed culture growth attesting to its use as primary carbon source. On the other hand, marked xylose consumption by strain ТMB3400 only occurred towards the end of the cultivation period (Figure 2C), with the magnitude of xylose consumption apparently not strongly correlated with the increase in biomass concentration. In fact, the data revealed that strain D5A^+H^ consumed 33% more xylose supplied to the culture than that consumed by strain TMB3400 (Table 3). This level of consumption was also reflected in the more than 7-fold greater biomass yield (Y_x/s_) on xylose by the latter strain (Table 3), which suggested that a carbon source other than xylose was preferentially utilised by strain TMB3400. Conversely, the low Y_x/s_ for strain D5A^+H^ might be attributable to ethanol production. Whereas no ethanol production was recorded for strain TMB3400 (Figure 2C) nor for strain D5A^+^ in the absence of liquor (Table 3), an ethanol concentration of 1.54 g/L was recorded for strain D5A^+H^ (Figure 2E), which pointed to increased efficiency in the conversion of xylose to ethanol, especially considering that the Y_x/s_ and Y_p/s_ values (biomass and ethanol on xylose) for strain D5A^+H^ were relatively similar (Table 3).

The by-product time profiles for strains D5A^+^ TMB3400 and D5A^+H^ are shown in Figure 2B, D and F, respectively. The largest decrease in by-products coincided with the exponential growth phase of strains D5A^+H^ and TMB3400. This inverse correlation was especially evident in the case of strain TMB3400 where a distinct biphasic profile was evident and could be related to the longer lag phase of this culture, which lasted for up to 42 h. The shorter lag phase of strain D5A^+H^ coincided with earlier commencement of by-product utilisation and/or detoxification. No significant decrease in either organic acids or furaldehydes was observed in the D5A^+^ control culture, attributable to the absence of culture growth (Figure 2A and Table 3).

Using glucose as carbon source, similar growth profiles were recorded for strains D5A^+H^ (Figure 3A) and TMB3400 (Figure 3C) in the presence of 50% (v/v) pretreatment liquor, which corresponded to that of the two reference strains, MH1000 and MEL2. The μ_max_ for strain D5A^+H^ in the presence of inhibitors was marginally greater than that of the other strains (Table 4), although a short lag phase of 4 h was apparent for this strain compared to the reference strains. The μ_max_ for the parental D5A^+^ strain was more than 20-fold lower than that recorded for strain D5A^+H^ (Table 4). Although the Y_x/s_ between the strains varied (Table 4), the Y_x/s_ value of strain D5A^+H^ was significantly greater than that of the other strains and could be related to the significantly greater biomass concentration recorded at the end of the exponential growth phase (Figure 3A, Table 4) and was corroborated by the significantly lower ethanol yield on glucose (Y_p/s_). In other words, strain D5A^+H^ clearly produced less ethanol relative to biomass (Y_p/x_), although the greater biomass concentration of this strain resulted in an ethanol titre similar to that of the other strains at the end of the exponential growth phase. Due to a severe lag phase in excess of 48 h recorded for the D5A^+^ parental strain, the data for this strain was omitted from Figure 3. The by-product profiles displayed in Figure 3B, D, F and H showed a substantial decrease in the acetic acid concentration for strains D5A^+H^ (50%), TMB3400 (27%), MH1000 (20%) and MEL2 (<10%), respectively. No clear pattern of formic acid removal was distinguishable for strains TMB3400, MH1000 and MEL2, whereas the formic acid concentration in the culture of strain D5A^+H^ decreased by more than 65% over the cultivation period.

Supplying either glucose or xylose as the primary carbon source distinctly affected the yeast metabolism, evident from the absolute differences in the fermentation parameters presented in Tables 3 and 4. The most prominent differences between strains D5A^+^ and ТMB 3400 are highlighted here. In the absence of pretreatment liquor with xylose as carbon source, the μ_max_ of strain D5A^+^ was 34% lower than with glucose, whereas the biomass yield coefficient was almost 7-fold greater. Ethanol production was absent with xylose as carbon source, whereas comparatively high levels of ethanol was produced on glucose. A similar response was evident for strain TMB3400 where the μ_max_ value on xylose was 36% lower than on glucose, with a substantially lower Y_x/s_ value on glucose (>8-fold difference), and complete absence of ethanol formation. Although these perturbations were less pronounced in the case of strain D5A^+H^, the most prominent difference was the ability of the latter strain to produce ethanol when xylose was the sole source of carbon. However, xylose was not as effectively utilised as a fermentable carbon source compared to glucose (Y _EtoH/Glc_ > > Y_EtoH/Xyl_).

### Strain performance during simultaneous saccharification and fermentation

**Figure 4:**
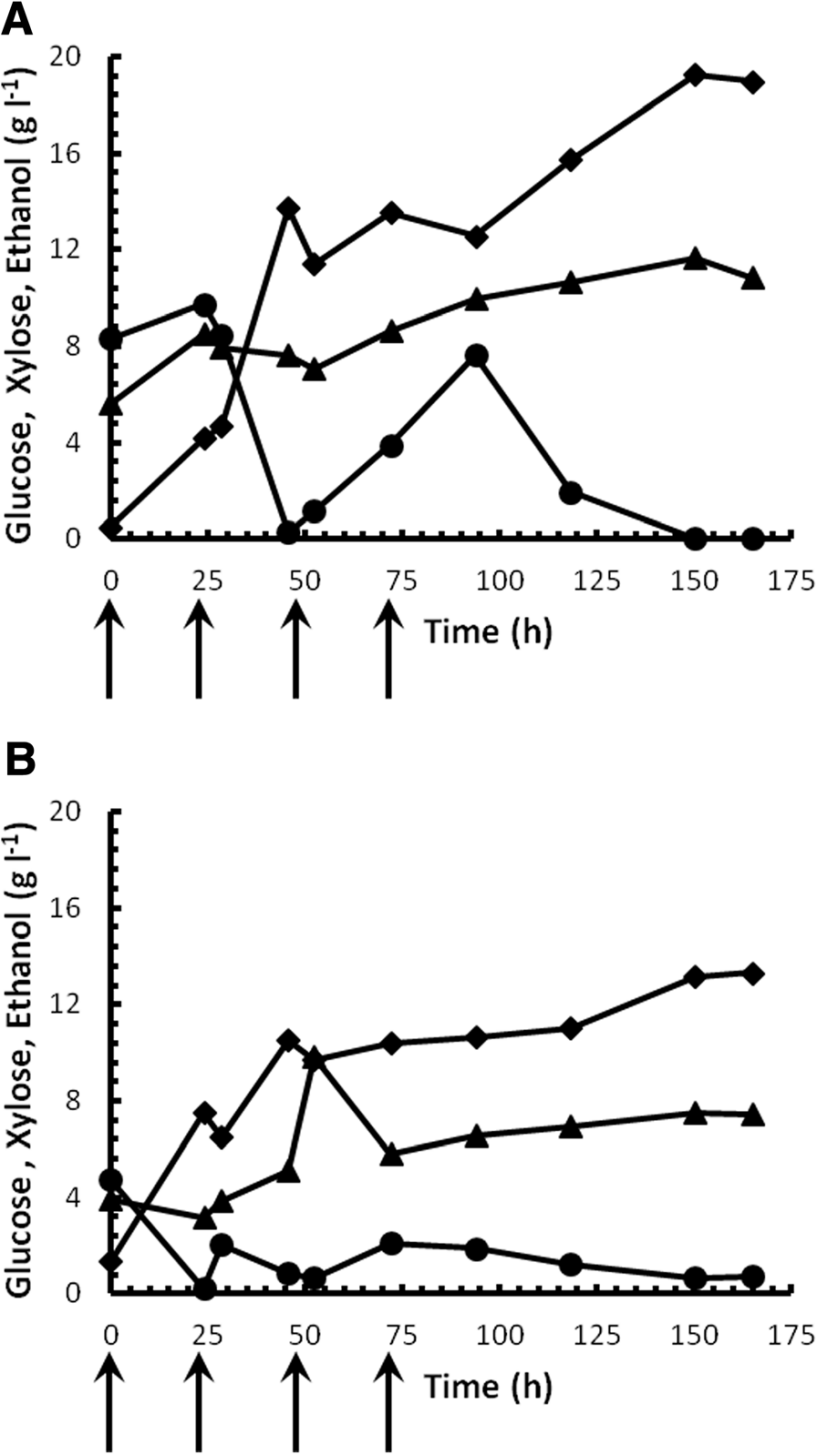
**Fed-batch SSF culture using steam-pretreated sweet sorghum bagasse.** Single fed-batch SSF experiments of pressed solids fed-batch using *S. cerevisiae* strains D5A^+H^**(A)** and TMB 3400 **(B)**. Figure symbols: ethanol (diamonds), glucose (circles) and xylose (triangles). Arrows indicate the addition of substrate in 2.5% (g/v) increments at 0, 24, 48 and 72 h.

Fermentation profiles for strains D5A^+H^ and TMB3400 during simultaneous saccharification and fermentation (SSF) are presented in Figure 4 using the water-insoluble solids (WIS) from steam-pretreated sweet sorghum bagasse as carbon source. Although it would have been preferable to continue with triticale solids we were restricted to using limited amounts of sorghum solids, whereas most hydrolysate from triticale was consumed during continuous culture experiments. Given that this section of the work was to provide a first order assessment of strain performance in SSF in the presence of inhibitors, we opted for an approach where triticale hydrolysate was used for hardening and sorghum was used for SSF work, in light of the relatively similar inhibitor concentrations in the hydrolysate from both feedstocks. Although the pretreatment liquor was separated from the WIS using a mechanical press, the solids were not washed prior to addition to the growth medium, which implied that a portion of the inhibitory by-products from the pretreatment liquor were carried into the SSF medium. A fed-batch fermentation strategy was employed to reduce broth viscosity and minimise exposure of the culture to high inhibitor levels, where feed times are indicated by the arrows.

On sweet sorghum bagasse solids, strain D5A^+H^ produced 45% more ethanol than strain TMB3400 reaching a maximum of 19.22 g/L after 150 h. Although this concentration equated to a very low 50% of the theoretical maximum, accounting for both glucose and xylose in the WIS and assuming a sugar to ethanol conversion factor of 0.51 g/g, this data served to illustrate the difference in the performance of the yeasts, since much room for SSF optimisation remains. For both strains, ethanol increased at the greatest rate before the third solids feed at 48 h, followed by a substantial decrease in the ethanol production rate. The profiles of residual glucose and xylose concentrations also differed distinctly between the two strains. For strain TMB3400, residual glucose levels remained consistently below 2 g/L throughout the fermentation, in spite of the addition of substrate at 24, 48 and 72 hrs. In the case of strain D5A^+H^, glucose accumulated to levels above 7.5 g/L directly after the addition of substrate at 24 and 48 hrs, after which consumption again commenced. The accumulation in residual glucose in the culture of strain D5A^+H^, and to a lesser extent with strain TMB3400, coincided with the levelling off of ethanol production. With strain D5A^+H^ a further increase in ethanol production again coincided with an increase in residual glucose consumption which occurred after approx. 96 h, whereas a less pronounced increase in ethanol production was evident for strain TMB3400 after 118 h. No residual glucose was detected in the culture of strain D5A^+H^ after 165 h, whilst a residual glucose concentration of 0.73 g/L was recorded in the culture of strain TMB 3400. This observation suggested strong recovery by strain D5A^+H^ from introduced toxic by-products after the third feed, especially in light of the final ethanol titres recorded in this culture, whereas the behaviour of strain TMB3400 remained more consistent throughout the cultivation. Xylose accumulated throughout the fermentation of both strains TMB3400 and D5A^+H^, although the final residual xylose concentration was more than 35% (7.5 g/L) lower for strain TMB3400 when compared to strain D5A^+H^ (11.6 g/L).

## Discussion

Two key strain development techniques, namely random mutagenesis and evolutionary engineering were sequentially used to harden a recombinant *S. cerevisiae* strain D5A^+^ against inhibitors from steam-pretreated triticale straw. Simultaneously, selective pressure through xylose as carbon source was imposed during both steps to enhance the metabolism of this pentose sugar via the XI pathway. Xylose isomerase represents the advantage of avoiding co-factor imbalances in the microbial metabolism at low oxygen tension, as opposed to the XR/XDH pathway, where XR was found to prefer NADPH as co-factor [11,24,42]. Furthermore, during evolutionary engineering, Koppram *et al*. [18] observed decreased xylose consumption by strain TMB3400 (XR/XDH system), apparently due to competition for reductive power between XR and furaldehyde. An expected outcome from the present study would thus be improved xylose fermentation to ethanol in semi-aerobic culture in the presence of pretreatment liquor from XI expression.

Given the complexity of the yeast metabolism on xylose as carbon source in the presence of inhibitors, random mutagenesis presented a more practical approach than rational metabolic engineering [15] to simultaneously enhance xylose fermentation and inhibitor tolerance. As such, we resorted to chemical mutagenesis using EMS where an expected outcome would be at least improved growth in the presence of hydrolysate inhibitors, using xylose as sole source of carbon compared to the parental strain as control. Evolutionary engineering for at least 100 generations at a high dilution rate (close to μ_max_) with xylose as sole source of carbon and in the presence of inhibitors from non-detoxified liquor would have the expected outcome of enhanced tolerance evidenced by an improved μ_max_ value, and ethanol yield and productivity compared to known benchmark strains. Therefore, there is a clear correlation between the developmental steps employed and the outcomes expected from the study, namely (i) xylose fermentation from metabolic engineering using XI, (ii) enhanced inhibitor tolerance and conversion from mutagenesis and evolutionary engineering, and (iii) a superior phenotype for ethanol production from xylose or glucose also from mutagenesis and evolutionary engineering.

### Hardening methodology

The high dilution rate in our chemostat culture supplemented with hydrolysate would allow for strong selective pressure in favour of yeast cells with improved xylose carbon flow through the metabolism. In this way cells with high growth rate would be selected. Most studies dealing with chemostat-based evolutionary engineering maintained continuous cultures at very low dilution rates in the region of D = 0.02 to 0.05 h^-1 ^[18,20,43]. Whereas low dilution rates are normally used to select for substrate affinity [14,44], the purpose in our study was to select for high growth rate, given the negative effect that inhibitors have on the culture μ_max _[8,45]. This approach corresponds to the more frequent use of sequential batch culture for evolutionary engineering, where the culture would grow at μ_max _[11,16-18,22,26,29,46,47]. Theoretically, evolved strains with the ability to maintain a higher growth rate should become dominant in a culture at a dilution rate close to μ_max_. An expected outcome would be an improvement in ethanol productivity, although this improvement was difficult to quantify in our work due to the absence of ethanol production by strain TMB3400 and the parental strain in xylose batch cultures (Table 3). The benchmark strains outperformed strain D5A^+H^ in terms of ethanol productivity in glucose batch cultures (Table 4).

### Strain performance with xylose as carbon source

An oxidative metabolism implies a greater biomass yield than would be the case with a respiro-fermentative metabolism and hence, a concomitant decrease in the ethanol yield [48]. This principle was patently evident from the data presented in Tables 3 and 4 where the Y_x/s_ value of the parental D5A^+^ strain on glucose without hydrolysate supplementation was more than six-fold lower than when xylose was supplied as carbon source in the chemically defined medium, which implied a predominantly oxidative metabolism with xylose as carbon source. These observations could, on the one hand, support the notion that *S. cerevisiae* fails to recognise xylose as a fermentable carbon source [49]. On the other hand, the strain D5A^+H^ produced 1.54 g/L ethanol in the presence of 50% (v/v) pretreatment liquor, whereas no measurable ethanol was produced by strain TMB3400 or by the parental D5A^+^ strain on xylose without pretreatment liquor (Table 3). Furthermore, strain D5A^+^ exhibited strong ethanol production from glucose at a yield of 0.42 g/g glucose (82% of the theoretical maximum), which still makes it a prime candidate for 2^nd^ generation ethanol production from cellulose. It is for the latter reason that the recombinant strain was subjected to mutagenesis and evolutionary engineering in an attempt to enhance xylose fermentation to ethanol, which was indeed accomplished. This result suggested that the mutagenesis treatment and/or evolutionary engineering profoundly affected the metabolism for funnelling carbon from xylose into the EMP pathway for ethanol production. The reprogramming of these pathways was described by Liu [50] and could possibly have been responsible for our observed result. Complete genomic analysis, coupled with transcriptomic analysis is earmarked for the future to elucidate the nature of this modification.

The negative impact of inhibitors on the ethanol productivity and yield [6,51] as well as on the biomass yield due to interactions between furaldehydes and acetic acid [52] is well described. Previous reports also illustrated that yeast has a diminished ability to deal with the toxic effects exerted by lignocellulosic inhibitors when grown on xylose as carbon source [53]. This theoretical basis might explain why the parental strain D5A^+^ in the present study was unable to grow and retain viable cell numbers on xylose in the presence of 50% (v/v) pretreatment liquor (Figure 2A). However, given this complete absence of growth of the un-adapted parental strain in the presence of inhibitors, the evolutionary engineering approach followed proved effective to introduce tolerance into the D5A yeast platform, as evident from the comparable μ_max_ values of strains D5A^+H^ and TMB3400 (Table 3). The fact that the μ_max_ of 0.12 h^-1^ of the adapted strain D5A^+H^ on xylose in the presence of pretreatment liquor (corresponding to the dilution rate close to the washout dilution rate in chemostat culture with 60% (v/v) pretreatment liquor) was 68% lower than that of the parental strain without hydrolysate clearly illustrated the toxic effect of the inhibitors on this yeast, in spite of the level of tolerance that was introduced into the yeast (Table 3).

Notably the μ_max_ value of strain TMB3400 recorded in the present study on xylose in the presence of pretreatment liquor was comparable to data published by Wahlbom *et al*. [10] for that strain in aerobic bioreactor cultures without pretreatment inhibitors. Therefore, compared to strain D5A^+H^ on xylose as carbon source, the presence of inhibitors appeared to have a less drastic effect on strain TMB3400, possibly attributable to the presence of XR. Furfural was shown to serve as an effective electron acceptor for XR, as evident from decreased xylitol formation, resulting in an improved ethanol yield [42], although no ethanol production by strain TMB3400 on xylose was observed in the present study (Table 3). Furthermore, strain D5A^+H^ exhibited a much shorter lag phase than strain TMB3400, where the lag phase for the latter strain lasted up to 30 h (Figure 2A, C and E). A long lag phase in the presence of inhibitors is a known occurrence, attributable to the time required for the cells to carry out detoxification [54,55]. Therefore, in spite of the inhibitory effect on strain D5A^+H^, the similarity of the growth rates between the two strains and the shorter lag phase pointed to our D5A^+H^ strain as a possible candidate for further development for industrial fermentations.

The *in situ* conversion of HMF and furfural by strain D5A^+H^ was clearly superior compared to the parental D5A^+^ strain and TMB 3400 (Figure 2B, D and F). Hence, HMF and furfural removal commenced immediately after inoculation due to a comparatively shorter lag phase, and similar to that reported by Palmqvist and Hahn-Hägerdal [8]. Previous reports identified a range of oxido-reductases as the primary enzymes responsible for the *in situ* reduction of HMF and furfural to their less toxic alcohol derivatives [56], such as the native reductases ADH6 and ADH7 [31,57-59]. One may, therefore, speculate on enhanced catalytic activity and/or higher degree of expression by one or more of these reductases brought on by mutagenesis and evolutionary engineering of strain D5A^+H^. Less than 12% of the xylose consumed by strain D5A^+H^ was converted to ethanol (Table 3), which implies accumulation of another metabolite, since the total recovered carbon from biomass, ethanol and glycerol was only 60%. Xylitol production has been observed in recombinant *S. cerevisiae* strains expressing XI and was ascribed to the endogenous activity of non-specific NADPH-dependent aldo-ketoreductases expressed from the *GRE3* gene [60,61]. Xylitol was not quantified during these fermentations, and future investigations will verify whether xylitol can account for these discrepancies in the carbon mass balance and transcriptomic profiling as well as enzyme activity data is required to validate this hypothesis.

The ability of strain D5A^+H^ to attain high growth rates and a shorter lag phase in spite of the presence of weak organic acids may point to a decreased sensitivity towards anion accumulation. The more abundant organic acids, i.e. acetic and formic acid, also play a pivotal role in contributing to pretreatment liquor toxicity [8]. However these acids are typically not reduced in a typically enzyme-mediated manner, as is the case for HMF and furfural [62], but rather accumulate intracellularly in dissociated form due to the neutrality of the cytosol [8]. The detoxification profiles show acetic and formic acid (Figure 2B, D and F) decreasing over time, with the fastest overall removal of these weak acids apparent for strain D5A^+H^. This observation could probably be related to earlier onset of cell growth. The greater biomass concentration probably resulted in faster diffusion of weak acids down its concentration gradient into the cytoplasm of the yeast cells. Although a large proportion of these anions were probably still secreted, this efflux was probably masked by increasing biomass and repetition of the cycle.

### Strain performance with glucose as carbon source

Compared to the parental D5A^+^ strain in the absence of pretreatment liquor, the influence of inhibitors on the cultures with glucose as carbon source was clearly evident from μ_max_ values that were up to 2.7-fold lower (strain MEL2) and q_p_ values that were up to 5.5-fold lower (strain D5A^+H^ Table 4). This data clearly illustrated the dramatic negative effect of inhibitors on productivity rates, whereas the biomass and ethanol yield was affected to a much lesser extent and corresponded to data reviewed by Liu [50]. Although strain D5A^+H^ surpassed strains ТMB 3400, MH1000 and MEL2 in terms of biomass yield and growth rate, this better performance did not correspond to higher ethanol productivity or yield during growth on glucose (Table 4). The comparatively higher Y_x/s_ value of strain D5A^+H^ on glucose could possibly be viewed as a secondary effect of mutagenesis and long term selection, since this trait was not observed in the parental yeast in the absence of pretreatment liquor. On the other hand, strains MH1000 and MEL2, which had comparatively lower μ_max_ values, produced ethanol at a significantly greater rate than strains D5A^+H^ or ТMB3400. This result is not surprising, given the robust nature of these yeasts, specifically strain MH1000, which was purposefully included due to its high ethanol productivity [41]. The by-product detoxification profiles showed that both acetic and formic acid (Figure 3B, D, F and H) decreased over time for all the strains. However, once again strain D5A^+H^ outperformed the other strains since approximately 20% more acetic acid was removed than was the case with strain TMB3400. This observation supports our previous suggestion of a decreased sensitivity to these acids was brought about through mutagenesis and evolutionary engineering.

### Practical implementation through simultaneous saccharification and fermentation

Whereas our results clearly demonstrated a degree of inhibitor tolerance by strain D5A^+H^, it is also of industrial importance to evaluate its effectiveness during SSF using steam-treated material. Therefore the ability of this strain was assessed along with that of the benchmark strain TMB3400 (Figure 4). A fed-batch approach was followed, to allow for a higher solids content of 10% (w/v), whilst minimising yeast inhibition [63], and viscosity and mass transfer effects. The fed-batch strategy should also further assist in xylose metabolism by limiting the concentration of residual glucose [63,64]. Strain D5A^+H^ performed substantially better than strain TMB3400 in SSF culture with pressed WIS, producing 46% more ethanol at a combined (glucose and xylose) theoretical yield of 50% vs. 34% for strain TMB3400. The data recorded for strain D5A^+H^ was slightly below that reported in the literature where ethanol yields from steam-pretreated sorghum ranged between 60-64% of theoretical maximum [65,66]. Although xylose was poorly utilised by strain D5A^+H^ in SSF culture, as evident from accumulation of residual xylose (Figure 4A), it is possible that the greater ethanol titre produced by strain D5A^+H^ could in part have stemmed from xylose, given the level of ethanol produced by this strain in batch cultures supplemented with hydrolysate and xylose (Table 3). Conversely, a possible explanation for the comparatively poor degree of xylose utilisation observed for the D5A^+H^ strain during SSF could be attributed to residual glucose levels that remained high for a large portion of the total fermentation time, which may have resulted in the inhibition of xylose consumption. High concentrations of glucose are known to inhibit xylose transport in yeast, due to competition between the two substrates for the hexose transporters which have an inherently lower affinity for xylose [67].

## Conclusions

The adaptation of yeast cultures for the production of ethanol from pretreated lignocellulosic substrates is of critical importance due to the toxic nature of the broth after pretreatment. Whereas other methods such as recursive breeding and genome shuffling may also be employed to beneficiate multi-allelic traits such as ethanol productivity and inhibitor tolerance [68], the experimental plan followed in this study yielded promising results. Therefore, we conclude that mutagenesis in combination with long term evolutionary engineering was successfully applied to introduce a greater level of tolerance in *S. cerevisiae* D5A^+H^, together with improved xylose fermentation. To the knowledge of the authors, this is one of very few studies where EMS treatment followed by evolutionary engineering at high hydrolysate concentrations with xylose as the sole source of carbon was successfully applied to a strain of *S. cerevisiae* harbouring the XI pathway for xylose consumption, resulting in comparatively superior performance to a known yeast benchmark. The physiological changes achieved in this study included an improved ability to ferment xylose in the presence of steam pretreated triticale straw liquor in batch culture. Future studies will include a second phase of long-term adaptation with emphasis on selection for increased ethanolic fermentation of xylose in the presence of pretreatment liquor, by keeping the fermentation broth strictly anaerobic. Additionally, genome sequencing as well as transcriptomic profiling of D5A^+H^ will be focused upon to elucidate the nature of the molecular mechanisms of tolerance inferred through the experimental approaches presented in this study, followed by directed mutagenesis at selected targets using a rational approach.

## Methods

### Triticale straw and sweet sorghum bagasse steam pretreatment

Triticale straw was kindly provided by the Department of Genetics at Stellenbosch University and sweet sorghum bagasse was obtained from the University of Kwazulu Natal both from South Africa. All biomass was steam-pretreated in a steam gun unit consisting of a 19 litre reactor vessel and a cyclone tank for material collection. Triticale straw biomass was soaked in water from reverse osmosis for a 16 h period in a 1:1 mass ratio after which excess water was removed from the material in a spin dryer for 5 min at 4000 rpm. Triticale straw was treated for 20 min at 200°C, whereas dry (i.e. not pre-soaked) sweet sorghum bagasse was treated for 5 min at 190°C. For both feedstocks, a solids loading of 500 g (dry weight) was used per steam gun run. Water insoluble solids (WIS) were removed from the slurry through compression in a 50 ton mechanical press at 25 kPa. The liquid and WIS fractions of the treated material were collected separately and stored at -20°C.

### Strains and maintenance

*Saccharomyces cerevisiae* strain D5A^*+*^ was used for performing random mutagenesis and evolutionary engineering. Strain D5A^+^ is a metabolically engineered xylose-utilising variant of the NREL D5A strain (ATCC 200062) and this recombinant strain as well as all other strains is deposited in the yeast culture collection of the Dept. Microbiology at Stellenbosch University, South Africa. Strain D5A^+^ contains a chromosomal integration of the putative xylose isomerase (*xylA*) gene from *Bacteroides thetaiotaomicron *[69]. The synthetic XI, which showed 83.1% identity with the *Piromyces* XI, was expressed from the multi-copy integrative vector pBKD1 together with the *XKS1* gene expressing xylulokinase from *S. cerevisiae* (R. den Haan, Stellenbosch University, unpublished results). S*accharomyces cerevisiae* TMB3400, employed as benchmark in this study, is a chemically induced mutant of the xylose utilising TMB 3001 strain, which is a CEN.PK derivative expressing xylose reductase (XR) and xylitol dehydrogenase (XDH) from the chromosomally integrated *Pichia stipitis* genes *XYL1* and *XYL2*, and over-expresses the homologous xylulokinase enzyme [10]. Two non-recombinant industrial isolates were also included, namely *S. cerevisiae* MEL2, which is a wild-type yeast isolated from grape marcs [40] and *S. cerevisiae* MH1000, which is a robust in-house yeast with a high fermentative capacity [41]. Strains where transferred from -80°C freezer stock cultures and routinely cultured on YPD (strains MEL2 and MH1000) and YPX agar plates (strains D5A^+^ and TMB3400), containing (per litre): 10 g yeast extract, 20 g bacteriological peptone, 20 g of either glucose or xylose and 15 g agar. All chemicals and reagents where obtained from Sigma Aldrich (Manheim, Germany).

The growth medium used for the cultivation of all yeast was based on the mineral medium described previously [70] and contained (per litre): 20 g yeast extract 0.5 g citric acid 3.4 g KH_2_PO_4_, 7.5 g (NH_4_)_2_SO_4_, 0.8 g MgSO_4_•7H_2_O, 0.05 g CaCl_2_•2H_2_O and 1 mL of a trace element solution, containing (per litre): 4.5 mg ZnSO_4_•7H_2_O, 0.3 mg CoCl_2_•6H_2_O, 1.5 mg MnSO_4_•H2O, 0.3 mg CuSO_4_•5H_2_O, 3 mg FeSO_4_•7H2O, 0.4 mg Na_2_MoO_4_•2H_2_O, 1 mg H_3_BO_3_ and 0.1 mg KI. All chemicals and reagents where obtained from Sigma Aldrich. Xylose and glucose were autoclaved separately at a concentration of 200 g/L and added aseptically after sterilisation to a final concentration of 20 g/L. To minimise evaporation of volatile by-products such as furfural, which precluded the use of autoclaving for sterilisation, pretreatment liquor was filter sterilised using a 0.45 μm nylon filter (Pall Corporation, NY, USA) and added aseptically to the medium. During continuous culture, the growth medium was supplemented with the anaerobic growth factors ergosterol and Tween 80 (Sigma Aldrich). Prior to addition to the growth medium, ergosterol was dissolved in 5 mL pure ethanol and subsequently added to this mixture. Final concentrations of ergosterol and Tween 80 in the growth medium were 0.01 and 0.42 g/L, respectively. Due to the sheer volume of hydrolysate required for continuous culture, the hydrolysate was not sterilised prior to feeding the culture.

### Random mutagenesis and primary selection culture

For mutagenesis the recombinant *S. cerevisiae* strain D5A^+^ was grown in batch culture using 50 mL of the chemically defined medium as described above, in a 250 mL Erlenmeyer flask supplemented with 20 g/L xylose as carbon source and incubated on an orbital shaker (New Brunswick Scientific, Edison, NJ, USA) at 30°C and 150 rpm. Flasks were fitted with foil-capped cotton plugs. Cells were harvested during the late exponential growth phase by centrifugation at 8 000 rpm for 5 min and re-suspended to a final concentration of 3.1 × 10^6^ CFUs/mL in phosphate-buffered saline (PBS) consisting of (per litre): 8 g NaCl, 0.2 g KCl, 1.44 g Na_2_HPO_4_, 0.24 g KH_2_PO_4_ and adjusted to pH 7.4 using 3 mol/L KOH. Chemical mutagenesis was carried out with ethyl methanesulfonate (EMS, Sigma Aldrich) according to the method described by Winston [71] using a 1 h incubation time on a rotating platform. EMS-treated cells were plated onto YPX agar, containing 20 g/L of xylose, and incubated at 30°C for 72 h to allow for colony growth of cells surviving the treatment. Untreated cells were plated on separate YPX plates as control. The percentage survival of mutants was calculated using the equation [*CFU*_*treated plate*_/*CFU*_*untreated control*_] × 100. In all cases cells were diluted using PBS. Relative to the control, the percentage survival of the mutated cells in the presence of 2, 3 and 6 μL EMS were 26 ± 2.3%, 21 ± 4.4% and 7.4 ± 1.2, respectively.

Colonies of surviving yeast treated with 2 and 3 μL EMS were washed from YPX plates with PBS, pooled and used as inoculum for selection of resistant mutants in medium containing pretreatment liquor. Two separate selection cultures at different pretreatment liquor concentrations where grown in batch culture in 500 mL Erlenmeyer flasks containing 100 mL medium. Whereas the one selection culture consisted out of the chemically defined medium (see above) supplemented with pretreatment liquor at a concentration of 67% (v/v), the other selection culture consisted of undiluted pretreatment liquor in which the dry powder of the medium components were dissolved. The growth of the mutated cells were followed over a six day incubation period at 30°C in a rotary shaker at 150 rpm and compared to that of the parental D5A^+^ strain which was grown under the exact same conditions and served as a control. Due to the dark coloration of the medium, cell growth was quantified with cell counts using an Improved Neubauer counting chamber/heamocytometer. For both the mutant and control strain, 20 g/L xylose was supplemented as the primary source of carbon.

### Continuous culture

Mutants that survived selection in the growth medium supplemented with 67% (v/v) pretreatment liquor were harvested by centrifugation after a six day cultivation period suspended in 50 mL PBS to a concentration of 72 × 10^7^ cells/mL and inoculated into a bioreactor for continuous culture. All fermentations were conducted in computer-controlled glass bioreactors (Sartorius Stedum BBI, Göttingen, Germany) with a total volume of 5000 mL and a working volume of 2000 mL. The bioreactor was equipped with a marine impeller, an exhaust gas reflux cooler perfused with water at 5°C, a pH electrode (Mettler Toledo, Halstead, UK) and a polarographic dissolved oxygen probe (Hamilton, Bonaduz, GR, Switzerland). Cultures were grown at 30°C and pH 5.0 by automatic titration with 3 mol KOH/L. Prior to inoculation of the reactor, the system was flushed with nitrogen to minimise oxygen levels at the onset of fermentation. To ensure anoxic conditions, no air was supplied to the reactor vessel during the six week cultivation which was operated at a stirrer speed of 200 rpm. Although neither air impermeable tubing nor a nitrogen overlay in the headspace of the culture was used, no measurable dissolved oxygen was observed throughout the cultivations. The culture was grown as batch culture for the first 48 h after inoculation before the feeding of the growth medium with hydrolysate commenced using a peristaltic pump (Watson Marlow Ltd., Falmouth, UK) adjusted to a flow rate of 50 mL/h to obtain a dilution rate of 0.05 h^-1^. To increase selective pressure, the dilution rate was gradually increased to a maximum of 0.125 h^-1^, which was in the vicinity of μ_max_ values determined in batch culture. At a dilution rate of 0.1 h^-1^, the hydrolysate concentration was increased from the original concentration of 50% (v/v) to a final concentration of 60% (v/v). The biomass concentration was estimated by total cell counts using an Improved Neubauer counting chamber. The large quantities of hydrolysate required for continuous culture (in excess of a total of 200 litres) precluded the use of filter sterilisation prior to addition to the bioreactor. Hence, the fermentation was performed under non-sterile conditions. Culture steady state was defined as a deviation in biomass concentration (cell counts/mL) of approximately 20% of the mean measured over three residence times. Although this variation is larger than what is usually acceptable, we allowed for a greater degree of error due to the harsh conditions impacting cell growth, the use of plate counts (not optical density), and limited hydrolysate availability, given a chemostat culture operated at a 2 L working volume. The total number of yeast generations was calculated by dividing the fermentation time by the cell doubling time at a given dilution rate.

### Strain isolation from continuous culture

At the end of six weeks of continuous cultivation the remaining yeast culture was harvested and subsequently purified through two rounds of sub-culturing on YPX agar. Culture purity was confirmed using microscopy. To verify that the isolated yeast was indeed a sub-strain of the parental recombinant D5A^+^ strain the isolated mutant was screened for resistance against the fungicide genetecin. A genetecin resistance gene was previously cloned into the parental strain for use as a selection marker during its genetic manipulation [69]. The adapted mutant (designated D5A^+H^) was found to be genetecin resistant, confirming that the isolated mutant yeast was related to the original parental D5A^+^ strain.

### Analysis of D5A^+H^ performance and benchmark comparison

Batch cultivations where performed in 500 mL Erlenmeyer flasks containing 100 mL growth medium consisting of the chemically defined medium (see above) supplemented with 50% (v/v) triticale pretreatment liquor. Glucose was supplied as the primary carbon source (20 g/L) for growth experiments using strains MH1000 and MEL2 whereas either glucose or xylose was supplied as a source of carbon (final concentration of 20 g/L) for the strains D5A^+^, D5A^+H^ and TMB 3400. The inoculum of each culture was prepared by growing the yeast for up to 36 h in a preconditioning medium consisting of the chemically defined medium (see above) supplemented with 20% (v/v) triticale hydrolysate. Batch cultures were inoculated at an initial OD value of 0.2, measured at 600 nm and incubated at 30°C on an orbital shaker at 150 rpm. Flasks were assumed to be semi-aerobic since cotton plugs were capped with aluminium foil.

### Simultaneous Saccharification and Fermentation (SSF)

All SSF experiments were conducted in the same bioreactors fitted with Rushton impellers and chemically defined medium as described above without supplemented carbon, implying that all carbon supplied to the culture was liberated by enzymatic hydrolysis from the steam-pretreated sweet sorghum bagasse. Nitrogen was sparged at a rate of 100 mL/min (0.05 vvm) to minimise oxygen availability. Due to the different temperature optima between enzymatic hydrolysis (45°C) and fermentation (30°C) a three hour pre-saccharification step at 45°C always preceded the SSF experiments. Yeast biomass was inoculated into the SSF medium to a final concentration of 5 g/L (wet mass). The inoculum for the SSF experiments was prepared using the same pre-conditioning method described in the previous section. Solids where loaded in 2.5% (w/v) increments at times 0, 24, 48 and 72 h to yield a final WIS solids loading of 10% (w/v) based on the total volume of the culture after the final WIS addition. A single dosage of cellulase was introduced at the onset of the pre-saccharification phase through addition of 0.667 mL Cellic® CTec2 (Novozymes, Denmark) per gram WIS. This volume was based on the same volume of Spezyme (Genencor, Palo Alto, CA, USA) that corresponded to approximately 15 FPU/g WIS, which was in excess of the required amount to achieve efficient hydrolysis of a 10% (w/v) solids loading of cellulosic material [72].

### Analytical methods

For the estimation of yeast biomass in g/L optical density measurements (absorbance measured at 600 nm) was correlated to yeast dry weight (g/L) through individual OD vs. dry weight standard curves for all strains used in this study. Dry biomass measurements were taken after fermentation samples were washed and vacuum filtered through a 0.22 μm Whatman filter paper and dried for a minimum of 12 h at 105°C. The chemical composition of the hydrolysate and water insoluble solids was determined according to the standard NREL procedures (LAP - 001 to LAP - 005 LAP - 010 and LAP - 017). Sugars, organic acids, ethanol and glycerol where separated on a Water Breeze (Waters Corporation, Milford, MA) high performance liquid chromatograph, fitted with an Aminex HPX-87H resin column (Bio-Rad Laboratories, Hercules, CA) and an H cartridge guard column (Bio-Rad). The column was operated at 45°C with 0.5 g/L H_2_SO_4_ as the mobile phase at a flow rate of 0.6 mL/min and detection was performed using a Waters 2410 refractive-index detector. HMF and furfural where separated on an Aminex HPX-87P column (Biorad). The column was eluted at 80°C with 0.5 g/L H_2_SO_4_ as the mobile phase at a flow rate of 0.6 mL/min and detection was performed using a Waters UV detector adjusted to 230 nm. Integration of the area underneath the peak of an eluted compound was relayed to concentration through the use of individual standard curves, based on standards injected at a range of concentrations prior to each run.

### Calculations

Maximum specific growth rates (μ_max_) where determined by linear regression of the slope of the natural logarithm of the biomass concentration plotted as a function of time using a minimum of four data points. The biomass (Y_x/s_) and product (Y_p/s_) yield coefficients were calculated by linear regression of the slope when biomass and ethanol where plotted as a function of the substrate concentration (either glucose or xylose) using a minimum of four data points recorded during the exponential growth phase. The specific rates of glucose xylose consumption (q_s_) and ethanol production (q_p_) were taken as the slope of the regression line fitted through the metabolite concentration vs. time profile normalised to the amount of biomass present at the midpoint of the exponential growth phase. Data was analysed on Microsoft Excel and imported into Design Expert v.1.6 (Stat-Ease Inc., MN, USA) for performing a one way analysis of variance (ANOVA), where a P-value of less than 0.05 was considered as statistically significant.

## Abbreviations

EMS: Ethyl methyl sulfonate; CFU: Colony forming unit; HMF: Hydroxymethyl furfural; SSF: Simultaneous saccharification and fermentation; WIS: Water-insoluble solids; XDH: Xylitol dehydrogenase; XI: Xylose isomerase; XK: Xylulokinase; XR: Xylose reductase.

## Competing interests

The authors declare have no competing interests to declare.

## Authors’ contributions

JS designed the experiments and carried out experimental work. EVR contributed to the experimental design, provided guidance for experimental work, especially continuous culture, and critically read and formatted the document. JFG provided critical inputs into the study in terms of experimental approaches and the content of the document. All authors read and approved the final manuscript.
